# Supplementary material for: Evaluation of the Conservation Status of the Croatian Posavina Horse Breed Based on Pedigree and Microsatellite Data
Source: Animals (Basel). 2021 Jul 18;11(7):2130. doi: 10.3390/ani11072130 (PMC8300408; doi:10.3390/ani11072130)
Supplement: Supplementary file 1 [file animals-11-02130-s001.zip › animals-1287074-supplementary.pdf]

CONTRIBUTION TO THE KNOWLEDGE OF CROATIAN POSAVINA HORSE

EXTERIOR DESCRIPTION

Croatian Posavina Horse is a breed with favourable interior qualities (i.e. calm temperament, good character, obedient and good-natured) with strong willingness to work. It is a medium size breed, firm constitution, and clean gait. The head is dry with broad forehead, flat profile, small ears, large and pronounced eyes and nostrils. The neck is of moderate length, well-muscled with a slight arching of the upper edge of the neck. The chest is broad and deep. The shoulder blades are long and oblique, well covered with muscle, firmly attached to the chest. The back is of medium length, strong, and broad, and the sacral region short and broad. The croup is broad, covered with muscles. The legs are dry and strong with well-defined joints. The *tibia* is short with firm joints and tendons. The fetlocks are poorly covered with short hairs. The conformation of the legs are mostly correct. The hooves are wide and of moderate hardness, adapted to movement on dry but also wet ground. The movements are impulsive, regular, firm with long stride. The mane and tail consist of dense, wavy and moderately long hair. Croatian Posavina Horse is always solid coloured, most often bay, less often black, chestnut, gray, yellow dun or isabell. Sexual dimorphism is pronounced. It is very adaptable to sometimes-unfavourable environmental conditions. It effectively utilises poor quality forage, therefore, it is suitable for an extensive husbandry system where it is grazed for most of the year with minimal supplementation of hay or green forage.

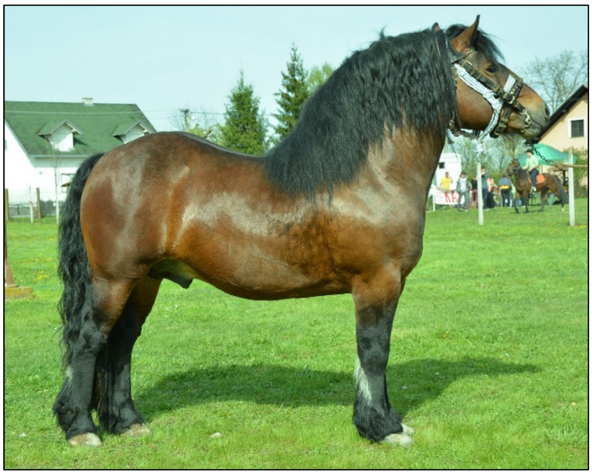

Figure 1. A stallion of Croatian Posavina Horse breed (*four years old*)

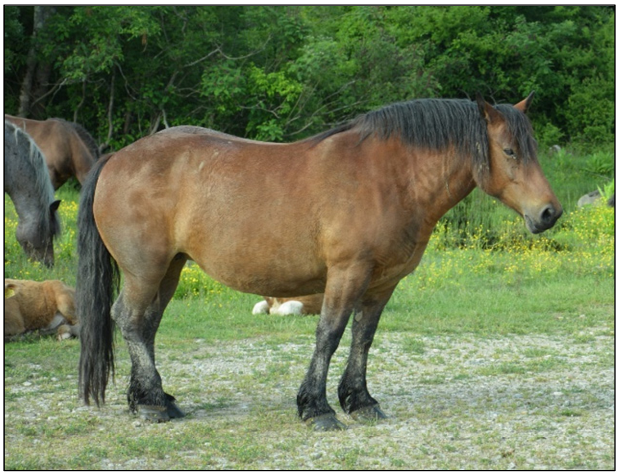

Figure 2. A mare of Croatian Posavina Horse breed on pasture (*six years old*)

BREEDING AREA

The breeding area of Croatian Posavina Horse consists mainly of the Sava River Basin (Sisak-Moslavina and Zagreb counties), which are not suitable for crops or other conventional agricultural production due to occasional flooding during autumn and winter periods. A small number of Croatian Posavina Horse is also bred in other parts of the Republic of Croatia.

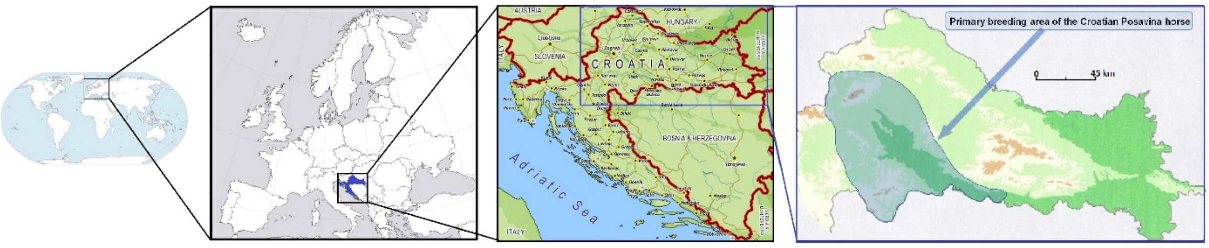

Figure 3. Primary (main) breeding area of Croatian Posavina horse

HISTORY OF BREED

Croatian Posavina Horse is a local horse breed that originated in Sava River Basin based on an archaic local horse population. It was created by systematic breeding of the local horse population (bušak) with controlled and limited introduction of warmblooded breeds (Arabian, Lipizzaner, Nonius), and, in the second half of the 20 century, coldblooded breeds. The archaic population (bušak) was suitable for agricultural and forestry work, driving, and pulling barges on rivers. Until the mid of the 20th century, the Croatian Posavina Horse belonged to archaic warmbloods horses. The agility and temperament of Croatian Posavina Horse indicate a significant influence of warmblooded ancestors, especially the Arabian horse.

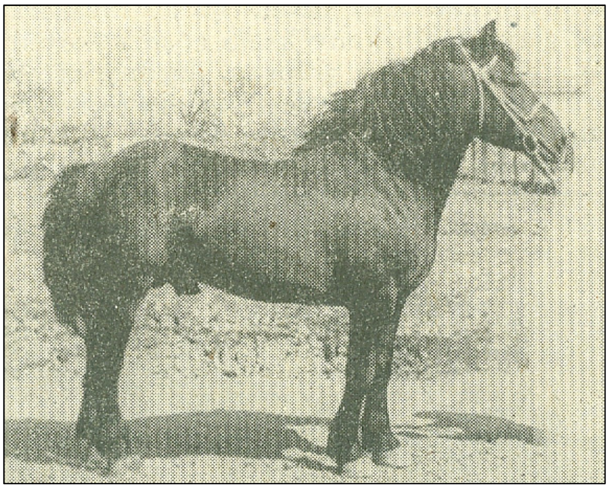

Figure 4. Stallion of the Croatian Posavina Horse in the middle of the 20th century [1]

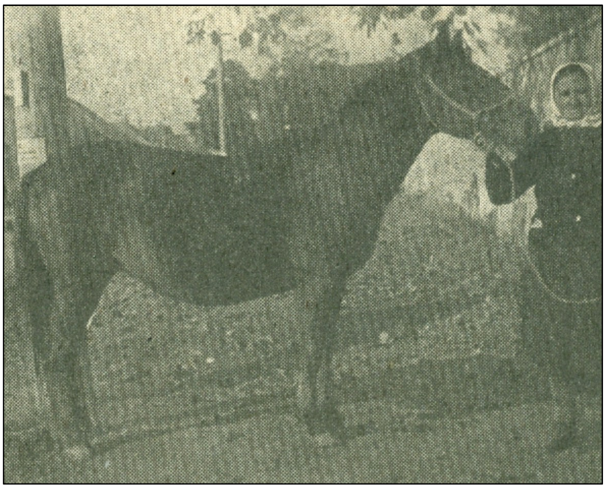

Figure 5. Croatian Posavina mare in the middle of the 20th century [1]

Ecological factors influenced the origin of the breed, as pastures, forests, and abundance of water made the rangelands suitable for year-round horse grazing. Steinhausz [2] states that the Croatian Posavina Horse is bred on moist and flooded pastures from early spring to late fall. During flood periods of the year, horses and foals often graze grass that emerges from the water. Climatic, pedological and hydrological factors have resulted in Croatian Posavina Horse becoming a horse with great adaptability to many negative environmental factors.

BASIC BREEDING DETERMINANTS

The principles of breeding and preservation of the Croatian Posavina Horse are defined by the Breeding Program [3] which are implemented by the Central Association of the Croatian Posavina Horse breeders (http://www.sshp.hr/). Only pure breeding is practiced and allowed.

The Studbook was established in 1998 and closed in 2003. There were 29 sire-lines, and 174 mare-lines established. The preservation of the sire-lines and mare-lines while respecting the breeding level in the relationship is the basis of the selection work and the preservation of the genetic diversity of the breed. Through the planned mating of sire-lines and mare-lines, an attempt is made to maintain genetic diversity, to avoid mating in close relationship, and selectively suppress undesirable characteristics of the breed from a breeding point of view. In breeding, the breed's adaptability is particularly appreciated (e.g. on wet and clayey soils, high temperatures, high humidity, etc.) as well as less demanding in terms of nutrition, especially compared to coldblooded horse breeds (e.g. Croatian Coldblood Horse breed).

ECONOMIC USE

Croatian Posavina Horse is economically utilised in the programme that assume producing foal meat of high quality. Reproductive activity is seasonal, from spring to late autumn. Breeders prefer the spring season. Mares foaled in the spring have enough pasture, and their foals growing fast while those foaled in autumn usually do not have enough pasture, they must be supplemented with hay and grains, and their foals grow slowly. Foals are weaned at six to eight months of age. Some of the male and female offspring remain for breeding purposes, and most foals are sold for meat production at about 12 months of age (from 9 to 15 months), when they reach a weight of 350 kg (from 300 to 400 kg live weight). The foals are mainly exported to neighbouring European countries, especially Italy. Only a small part of foal meat is placed on the Croatian market, as there is no habit of consuming horse meat. The price of foals destined for meat production is favourable (≈ 3 EUR / kg live weight), which makes breeding Croatian Posavina Horse in an extensive husbandry system profitable. In addition to foal meat production, Croatian Posavina Horse is used for enrichment of agro-tourism, folklore events, recreational activities, and to a lesser extent in agricultural activities.

It is important to note that Croatian Posavina Horse is predominantly bred in a traditional way which is basically extensive. That is, in early spring (February – April), horses are released to pasture, where they remain until late fall (October – November), with minimal supplemental feeding of hay or grain. In late autumn, herds of Croatian Posavina Horses are housed in simple barns where they remain for about three months. While stabled, the horses are fed by hay and modest amounts of grain. Such a traditional form of husbandry is favourable and sustainable in the long term, considering the relationship between costs and yields. A prerequisite for high-quality horse meat production is the reproductive activity of mares and stallions, which maintains the overall vitality of the population.

IMPORTANCE IN TRADITIONAL AND CULTURAL EVENTS

Croatian Posavina Horse plays a major role in preserving the traditional habits and way of life the region where it has been bred for centuries. Today, the carriage ride with Croatian Posavina Horse is an integral part of all cultural and folklore events in the counties where the breed is most represented (Sisak-Moslavina County, Zagreb County).

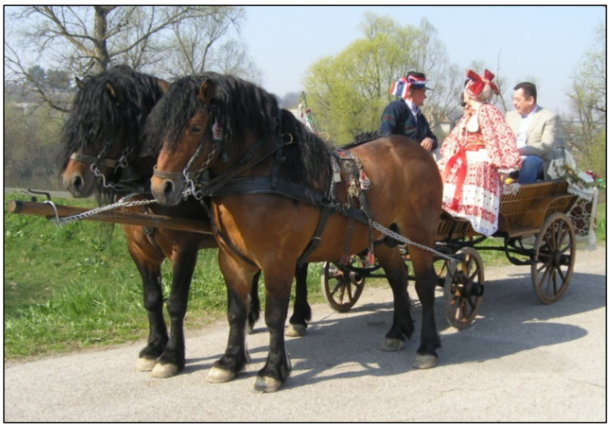

Figure 6. Traditional carriage with Croatian Posavina Horse

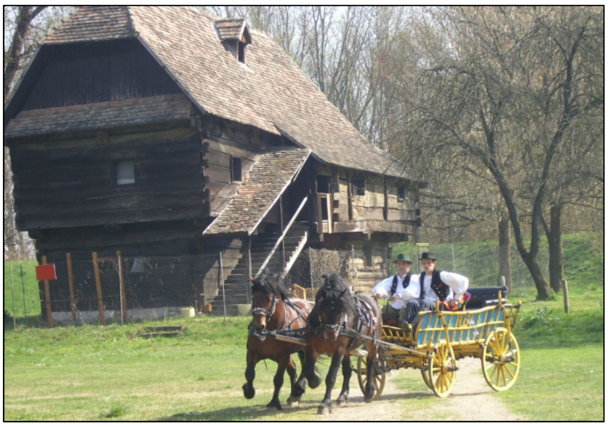

Figure 7. Carriage with Croatian Posavina Horse next to the traditional wooden „Posavina House"

ECO-SERVICE FUNCTION IN ECOSYSTEMS PRESERVATION

Croatian Posavina Horse is bred mainly in the Posavina area, which is characterized by wetlands, periodically flooded pastures, and lowland forests with oak, field ash, willow, alder, and poplar. The great importance of Croatian Posavina Horse lies in the conservation of floodplain habitats. The richness of flora and fauna makes this area unique on a regional and global scale, to which the traditional way of using the grazing resources also contributes. Herds of Croatian Posavina Horse fulfil a permanent eco-service function in ecologically sensitive areas.

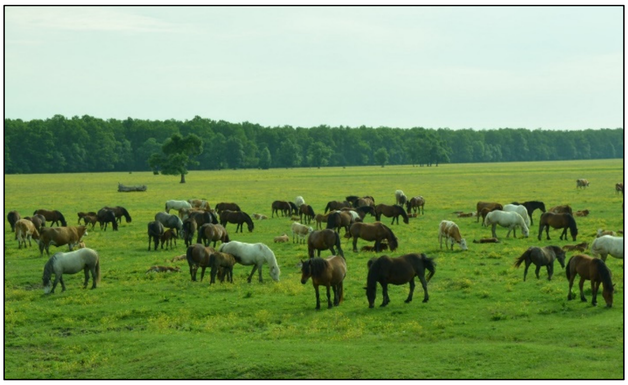

Figure 8. Croatian Posavina Horse with cattle on the pasture in Lonjsko polje

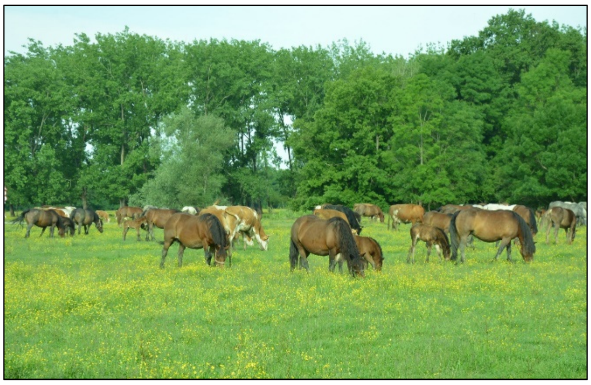

Figure 9. Herd of Croatian Posavina Horse on the pasture of Odransko polje

References

1. Ogrizek, A.; Hrasnica, F. *Specjalno stočarstvo. I dio. Uzgoj konja*. Poljoprivredni nakladni zavod: Zagreb, Hrvatska, **1952**, pp. 234–236.
2. Steinhausz, M. Uzgoj konja u Savskoj Banovini. *Izdanje kr. banske uprave Savske banovine u Zagrebu*, Zagreb, Hrvatska, **1935**.
3. Breeding program of the Croatian Posavina Horse, *Central Breeders Association of Croatian Posavina Horse*, **2019** (available on: https://www.sshp.hr/regulativa/uzgojna-dokumentacija/)
